# Radio Frequency Identification (RFID) in health care: where are we? A scoping review

**DOI:** 10.1007/s12553-022-00696-1

**Published:** 2022-08-23

**Authors:** Laura Profetto, Monica Gherardelli, Ernesto Iadanza

**Affiliations:** 1 Department of Information Engineering, University of Florence, Via di S. Marta, 3, Florence, 50139 Tuscany Italy; 2 Department of Medical Biotechnologies, University of Siena, via Aldo Moro, 2, Siena, 53100 Tuscany Italy

**Keywords:** RFID, Healthcare, Medical devices, Equipment, Management, Logistics

## Abstract

**Purpose:**

(RFID) is a technology that uses radio waves for data collection and transfer, so data is captured efficiently, automatically and in real time without human intervention. This technology, alone or in addition to other technologies has been considered as a possible solution to reduce problems that endanger public health or to improve its management. This scoping review aims to provide readers with an up-to-date picture of the use of this technology in health care settings.

**Methods:**

This scoping review examines the state of RFID technology in the healthcare area for the period 2017-2022, specifically addressing RFID versatility and investigating how this technology can contribute to radically change the management of public health. The guidelines of the Preferred Reporting Items for Systematic reviews and Meta-Analyses (PRISMA) have been followed. Literature reviews or surveys were excluded. Only articles describing technologies implemented on a real environment or on prototypes were included.

**Results:**

The search returned 366 results. After screening, based on title and abstract, 58 articles were considered suitable for this work. 11 articles were reviewed because they met the qualifying requirements. The study of the selected articles highlighted six matters that can be profitably impacted by this technology

**Conclusion:**

The selected papers show that this technology can improve patient safety by reducing medical errors, that can occur within operating rooms. It can also be the solution to overcome the problem of the black market in counterfeiting drugs, or as a prevention tool. Further research is needed, especially on data management, security, and privacy, given the sensitive nature of medical information.

**Graphical Abstract:**

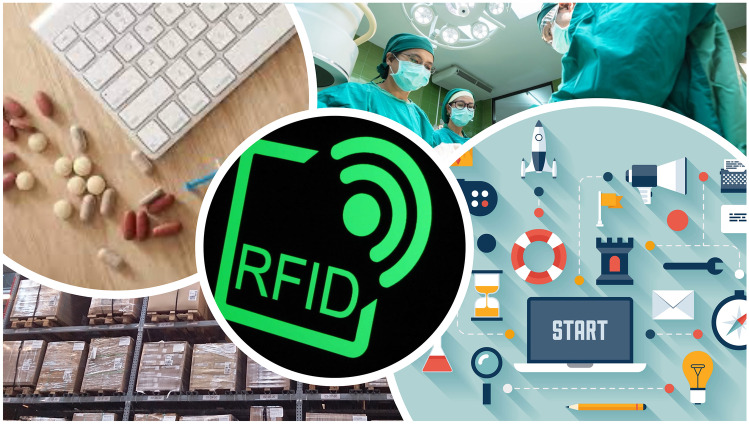

## Introduction

Today, the most important challenges for healthcare professionals are minimizing the impact of adverse events and improving patient safety [1]. An adverse event is defined as any complication that arises during the patient’s stay in hospital and is not directly related to the underlying disease or reason for hospitalization [2]. These events can have serious consequences for the patient, her / his family and even the health system. The concept of traceability can provide many benefits to these processes. Traceability means the identification of all information relating to a product from origin to delivery and / or consumption [3]. In the context of health services, this can be translated as the exact identification of the patient, the drug and the patient / drug relationship administered, which can significantly reduce the incidence of adverse events, thus increasing safety. Healthcare is currently facing the challenges of improving this aspect and reducing operating costs, which unfortunately are often caused by human and systematic errors. The American Institute of Medicine (IOM), recently renamed as the National Academy of Medicine (NAM) [4], estimated that between 44,000 and 98,000 deaths per year are related to medical errors occurring in hospitals, thus showing the desperate need to improve patient safety and well-being in hospitals [5]. It is possible to identify common phenomena that lead to serious healthcare operation failures in addition to medical mistakes, such as theft loss, and drug counterfeiting [6].

RFID technology is becoming more prevalent across a variety of industries, with the healthcare sector being a growing area. Indeed, the maturation of applications such as real-time locating system (RTLS) for patient tracking, medical personnel, and asset tracking will most certainly contribute to rapid expansion in the RFID industry in the future years. This market was worth USD 16.95 billion in 2016 and is expanding at a 7.7 percent CAGR between 2017 and 2023 [7].

Radio frequency identification (RFID) is one of the 16 fundamental innovations for the next decade, as stated by the Massachusetts Institute of Technology (MIT) which ranked it as the 10th most innovative technology of the last 25 years, for automatic data collection and traceability of goods [8]. The identification process consists in reading an RFID tag applied to an asset or a person without any physical contact. The data collection and transfer are done with the use of radio waves, so data is captured efficiently, automatically and in real time without human intervention. The advantage is that an RFID reader can read more tags simultaneously from a greater distance and therefore without the need to approach the reader, unlike traditional barcode scanning. It is therefore possible to attribute an electronic label to assets, healthcare personnel or patients, who once tagged, can be identified, tracked, and managed through a centralized database, using pervasive IT devices such as PDAs (Personal Digital Assistants) or mobile phones [9].

**Fig. 1:**
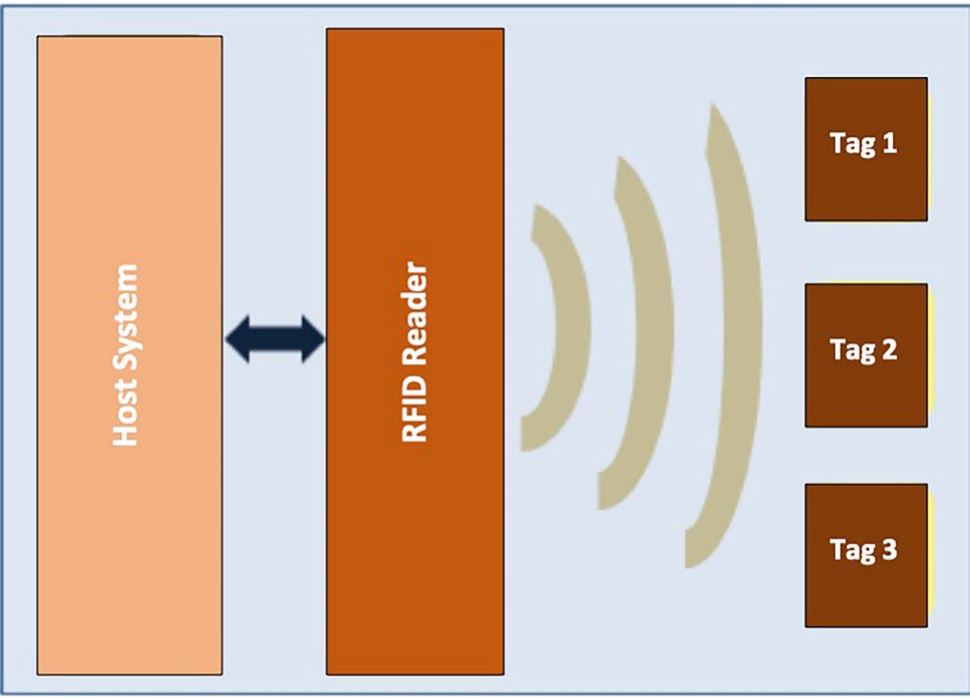
RFID system: an RFID reader acquires information from one or some tags and transfers such information to an host system

A RFID device can have different electromagnetic transmission configurations, based on different applications, but typically includes the following components (Fig. 1):

- Tag RFID;
- Tag reader equipped with an antenna and a transceiver;
- Host system or connection to a business system.

The Tag is used to store information; each RFID tag contains an electronic integrated circuit and an antenna inside a package (capsule), which are affixed to an object with a unique identification number and a memory that records additional data relating to the manufacturer, the product type and other related environmental information [10]. The reader is used to collect all the information stored in a tag. The RFID reader consists of a decoder that decodes the information; the antenna is used to transmit and receive the RF waves that carry information from the tag to the reader and *vice versa*. The RFID reader can read or/and write data in the tags by reading the identifying information (IDs) of the neighboring tags and mapping them to an object through a database or an external service. The software is used to manage the received data and the reader and tag operations, it manages the information in a database [10]. The latter can also contain the details of the tags and readers. All information is sent to a host computer or RFID middleware to ensure communication between the RFID infrastructure and the various intra- and inter-organizational systems [7]. Tags can be classified into three classes: active, passive and semi-passive tags [10].

Active tags are powered by batteries and incorporate both a receiver and a transmitter, have large memories, often rewritable, and can contain sensors. They can operate at distances that are generally much greater than those of passive and semi-passive tags (maximum 200 meters) and have larger memory. The disadvantages are: high price, limited duration as they depend on the antenna and the energy available in the batteries, larger weight and dimensions than passive tags.

Passive tags do not have an internal power source, they are activated when they enter the range of action of an RFID reader, the latter generates a magnetic field that powers, and therefore activates, the chip contained in the tag. Passive tags are smaller in size, lighter in weight and low in cost and with an unlimited lifespan. Unfortunately, they have limited functionality: they have a low communication range, their information storage and computing capacity is limited.

The semi-passive tag is provided with a battery that is used only to power the internal circuit. Unlike the active tag, it communicates via the electromagnetic field created by the reader. The battery stays dormant until triggered by a signal from the reader, saving battery power and extending tag life [7].

The RFID technology can operate at different frequencies, each having its pros and cons. For the low frequency (LF) band, 125 to 134 kHz, the main advantage is the possibility of its use worldwide, indeed it is available in all major countries: Europe, North America, and Japan. The major applications related to its use are those that require the transmission of limited amounts of data over short distances. It is also affected by small interference with liquids and metals [11]. The main drawback is that ferromagnetic materials have a shielding effect on electromagnetic waves at these frequencies and therefore can cause reading problems. Furthermore, the large dimensions of the reader antennas and the reduced operational distances limit the diffusion of systems using these frequencies [12].

The high frequency (HF) band has a central frequency of 13.56 MHz, and is characterized by greater reading range and speed than the LF band. Near Field Communication (NFC), a wireless data interface between devices also works at an operating frequency of 13.56 MHz [13].

The ultra-high frequency (UHF) band is between 860MHz and 960MHz. These tags have better reading range and better data transfer compared to lower frequency bands. Increasing the frequency allows the use of smaller antennas, that are therefore suitable for portable devices. On the other side, costs are higher with this technology. Usually the different governments, through their legislation, independently manage frequency assignments. Therefore, there are differences internationally in the frequencies assigned for RFID applications even if standardization by ISO and similar organizations is helping to make them more and more compliant. For example, Europe uses 868MHz for ultra-high frequency (UHF), while the United States uses 915 MHz [7].

The RFID technology used with other technologies, such as the Wireless Sensor Network (WSN), allows to expand its functionality and create hybrid monitoring systems, based on the Internet of Things (IoT) [7]. This hybrid technique depicts a possible progression of Internet use: objects (“things”) become recognized and intelligent since they can communicate their own data and receive aggregate information from others; as a result, all items can play an active role owing to Internet connectivity [14].

“Things” or “objects” are elements such as, among others: devices, instruments, plants and systems, materials, products, works, goods, machines, and equipment. The connected objects, that are the basis of the IoT, are more properly defined as “smart objects” and are characterized by some properties or functionality. Identification, connection, localization, the ability to process data and the ability to interact with the external environment are paramount [14].

The IoT is a system consisting of three levels [15]:

- Perception layer: also called “physical layer”, which identifies and collects all types of information from the physical world of the IoT, through sensors, tags, WSNs, cameras, RFID systems and so on.
- Network layer: also called “transport layer”, in charge of transparent data trasmission.
- Service layer: also called “application layer”, including a sub-level for data management and a sub-level for application services.

RFID technology, alone or together with other technologies, has been considered as a possible solution to reduce problems that endanger public health or for improving the management of the latter. For example, the problems related to medical waste recycling, if not managed in a safe and conscious way can cause the spread of diseases and environmental pollution, that traditional methods often fail to prevent. There are some studies aimed at finding a solution to this type of problems; some of these aim to design methods that apply reverse logistics based on RFID technology [16].

This work examines the state of RFID technology in the healthcare area in the last five years, It specifically illustrates RFID versatility and verifies how this technology can contribute to radically change the management of public health. The aspects that have an impact on the qualitative characteristics of health services relating to prevention, diagnostics and monitoring of patients’ health are considered very important.

## Methods

For this work it has been chosen the Scoping Review research design to assess the current state of RFID employment in healthcare area, to have an overview of the state of the art relating to the chosen topic and to identify the problems that limit RFID use. Scoping Review is a type of research evidence synthesis that aims to detect the literature on a particular research topic or area and to provide an opportunity to identify key concepts [17]. The guidelines of the Preferred Reporting Items for Systematic reviews and Meta-Analyses (PRISMA) have been followed. PRISMA statement aims to provide a guide for the drafting of the results of research in the medical field [18].

### Eligibility criteria

According to the selected eligibility criteria, only journal articles with a publication year from 2017 to 2022 were included. The examination of these articles, in particular, allows us to concentrate on newly created approaches, and the research confined to the aforementioned time allows us to comprehend the major elements and associated constraints of the most current methodology. The search was restricted to documents in English, which was thought to be the most often used language for this type of topic. Literature reviews or surveys were excluded. Only articles describing technologies implemented on a real environment or on prototypes were included.

### Searching for a paper

The searching of articles was carried out through Scopus, a search engine with a database of peer-reviewed scientific products (journal articles, books, conference proceedings) and more than 70 million bibliographic citations, abstracts, and bibliometric data. It was preferred over other search engines, as it covers wider disciplinary sectors, unlike for example Pubmed which is a purely biomedical database.

The search string launched on Scopus was as follows:

TITLE-ABS-KEY ( rfid AND ( healthcare OR “health care” OR hospital ) ) AND ( LIMIT-TO ( DOCTYPE , “ar”) OR LIMIT-TO ( DOCTYPE , “ch” ) OR LIMIT-TO ( DOCTYPE , “re” ) ) AND ( LIMIT-TO ( PUBYEAR , 2022 ) OR LIMIT-TO ( PUBYEAR , 2021 ) OR LIMIT-TO ( PUBYEAR , 2020 ) OR LIMIT-TO ( PUBYEAR , 2019 ) OR LIMIT-TO ( PUBYEAR , 2018 ) OR LIMIT-TO ( PUBYEAR , 2017 ) ) AND ( LIMIT-TO ( LANGUAGE , “English”) )

With this string we have imposed restrictions on the year of publication (from 2017 to 2022), and on the language: English.

### Selection process

**Fig. 2:**
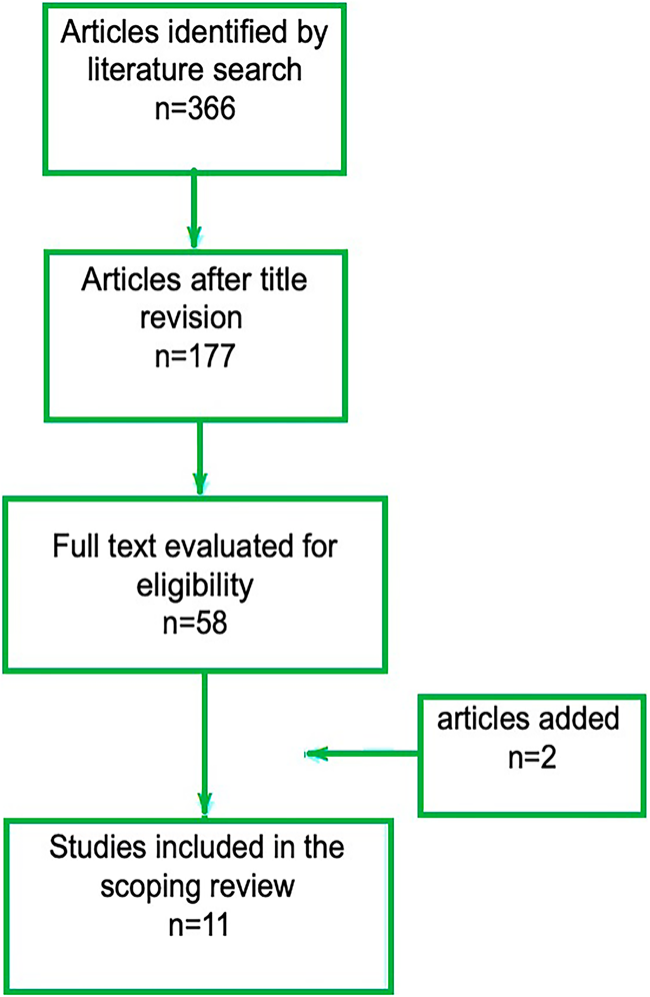
Selection process of papers

After the literature search, all the recovered documents were examined, selected first by title, then by abstract and finally by evaluating the entire text. The articles rejected based on their title were not related to the health sector but dealt with other issues. In reading the abstract, those articles relating to literature reviews or surveys were discarded. Articles with higher numbers of citations were preferred in this phase. All of the records in the output of the literature search had their titles and abstracts reviewed separately by two reviewers (L.P. and E.I.). The ones deemed to be unrelated to the scope of the review have been eliminated. The two reviewers’ individual results have been compared, and the publications that they both deemed appropriate for the research have been added immediately to the list for full-text download. M.G., a third reviewer, was requested to make a choice about the papers that had been chosen by just one of the two reviewers. The selection process of the sources of evidence is illustrated by means of the flowchart in Fig. 2.

## Results

The search returned 366 results. One paper was found among the references of [19] and was manually added to the final list [20]. An article concerning the cognitive learning of autistic children was also manually added. This paper deals with the guidelines for preventing Covid-19 infection and is the updated version of a paper [21] published in the Proceedings of the 12th Asian Conference on Intelligent Information and Database Systems [22]. Eleven articles were included in this review at the end of the selection process.

### Characteristics of sources of evidence

Tables 1 and 2 provide an overview of the selected articles. For each study, the reference, the used technology, the objective, advantages, limitations and the date of publication are indicated.

**Table 1:** Selected articles

Reference	Technology	Objective	Advantages	Limitations	Year
[14]	RFID (840 - 960 MHz)	Monitoring of medical equipment and drugs based on IoT technology	Product identification by means of the drug packaging, name, model, quantity, expiration date, etc.	Improvement in data security, this scheme is intended only for management following the drug purchase	2022
[23]	RFID	Infection prevention and control	Cognitive learning, addressed to autistic children, of precautionary actions guided by WHO to avoid the risk or spread of COVID-19	Limited number of participants to the evaluation study, children under the age of 4	2022
[24]	Passive Tag RFID (902 - 928 MHz)	Protective measures	Rollover detector for wheelchairs with warning to nearby hospitals and / or relatives	Improvements in sensor accuracy are needed.	2021
[25]	Passive Tag RFID (800 MHz-1 GHz antenna)	Prevention and control of Infection	Nappy moisture sensor	Possible improvements in sensor accuracy	2020
[26]	Passive Tag RFID	Prevention and control of Infection	Contact tracing for COVID-19 and other infectious diseases	Cost and safety	2020

**Table 2:** Selected articles

Reference	Technology	Objective	Advantages	Limitations	Year
[27]	Tag RFID	Monitoring of medical equipment and drugs	Identification of the product item through batch, serial number, expiration date; a closed cabinet (Faraday cage) protects products from electronic interactions	Tested in a confined environment	2019
[28]	Tag RFID (13.56 MHz)	Reduction of medical errors in the operating room	Monitoring of instruments during the surgical operation	The RFID tags are not designed to be applied to small instruments and require further tests	2018
[2]	Tag NFC (13,56 Mhz)	Patient identification	Tracing of hospitalizations, care plans, prescriptions and drug administrations	High costs and need to be more tested	2018
[20]	RFID	Remote and real-time monitoring of vital signs	Warning signal to doctors or family members in an emergency	Bulky sensors with limited storage capacity; limited device battery	2018
[29]	RFID	Infection prevention and control	Blood pH detection to monitor the state of a wound	More studies needed to improve flexibility	2017
[30]	Passive Tag RFID (13.56 MHz)	Reduction of medical errors in the operating room	Identification of surgical gauze	Further clinical studies needed to confirm reliability and applicability	2017

### Summary of results

The study of the selected articles highlighted six matters that can be profitably impacted by this technology.

#### Reduction of medical errors in the operating room

**Fig. 3:**
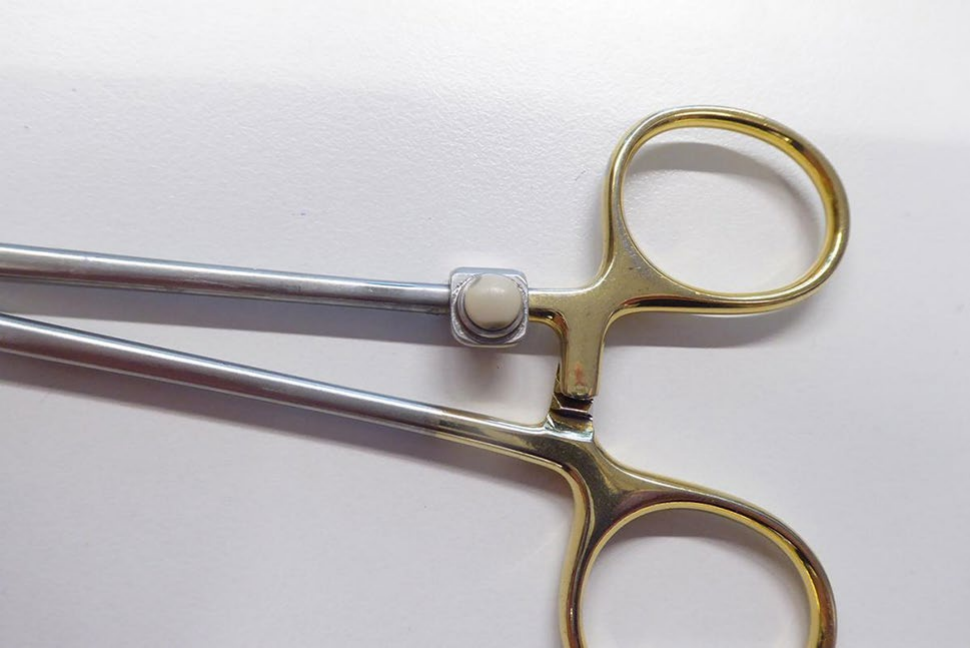
Radiofrequency identification-tagged instruments (source: https://www.delta.tudelft.nl/article/tracking-surgical-instruments-rfid-chips)

One of the most frequent adverse events related to the use of devices in surgery is the retention of surgical instruments, such as gauze (clinical condition defined in the literature as “Gossypiboma” or “textiloma”) needles, scalpels, electrosurgical adapters, forceps, or parts thereof. A wide range of clinical outcomes, including asymptomatic patients, cases with major consequences such intestinal perforation, sepsis, organ damage, and even death, can result from the retention of foreign material. Due to these events, a mortality rate of 11 % to 35 % is estimated [31]. Despite the refinement of the guidelines for equipment counting in surgery, the risk of retaining foreign objects is high, and can increase in some situations, such as during emergency operations with unplanned procedure or in the case of patients with a high body mass index (BMI). Therefore, the need to find a solution that can solve this problem at the root. Indeed, RFID technology has proven to be a reliable tool for detecting and tracking surgical material. For example, as regards the gauze, a system has been developed that includes an integrated antenna, capable of scanning the patient’s body and identifying the retained gauze. Each gauze is equipped with a passive RFID tag, in bio-compatible material, that is resistant to water, chemicals and high temperatures [30]. The count of the used gauze is carried out through a basket-shaped ‘check-out’ antenna, which consists of an array of six antennas: four on the side surface, one on the bottom and one at the intermediate level. The localization of the gauze is carried out through a multiplexer that acts as a body scanner. All data is displayed in real time with software supporting the operating room staff. Surgical instruments (scalpels, probes, hemostatic tissue, forceps, etc.) can also be identified with an RFID tag (Fig. 3) by using an antenna that is able to detect them and monitor their usage rate. The usage rate is an important parameter for understanding wear, thus preventing breakage of the instrument during surgery. The antenna is positioned on an instrument holder, the Mayo table, where the instruments are sorted and collected thus allowing a precise reading of the objects that are positioned above [28].

#### Patient identification

Misidentification of the patient is one of the main causes of medical error, leading to incorrect administration or incorrect dosage of drugs. These mistakes can lead to serious consequences. RFID technology has the potential to prevent such consequences. An example is the use of NFC tags to identify medical staff on shift, hospital patients and drugs [2].

**Fig. 4:**
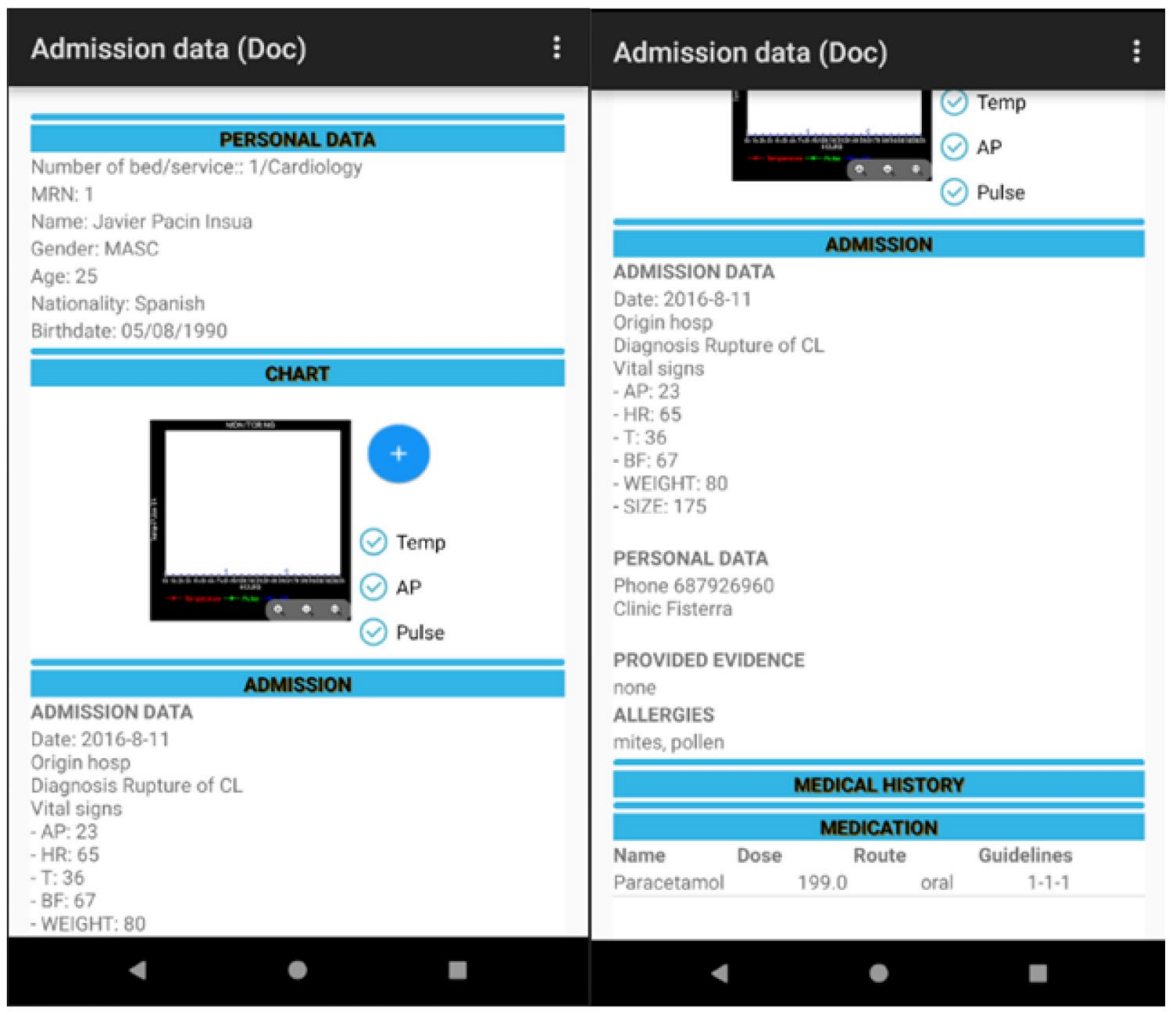
RFID system for tracking in the ICU (doctor (source: https://www.mdpi.com/1424-8220/18/5/1627/htm#)

In the Intensive Care Unit (ICU) of the Virxe da Xunqueira hospital in Spain, an interesting system has been implemented that computerizes and keeps track of hospitalizations, care plans, vital monitoring, prescriptions, and drug administration of patients (Fig. 4).

**Fig. 5:**
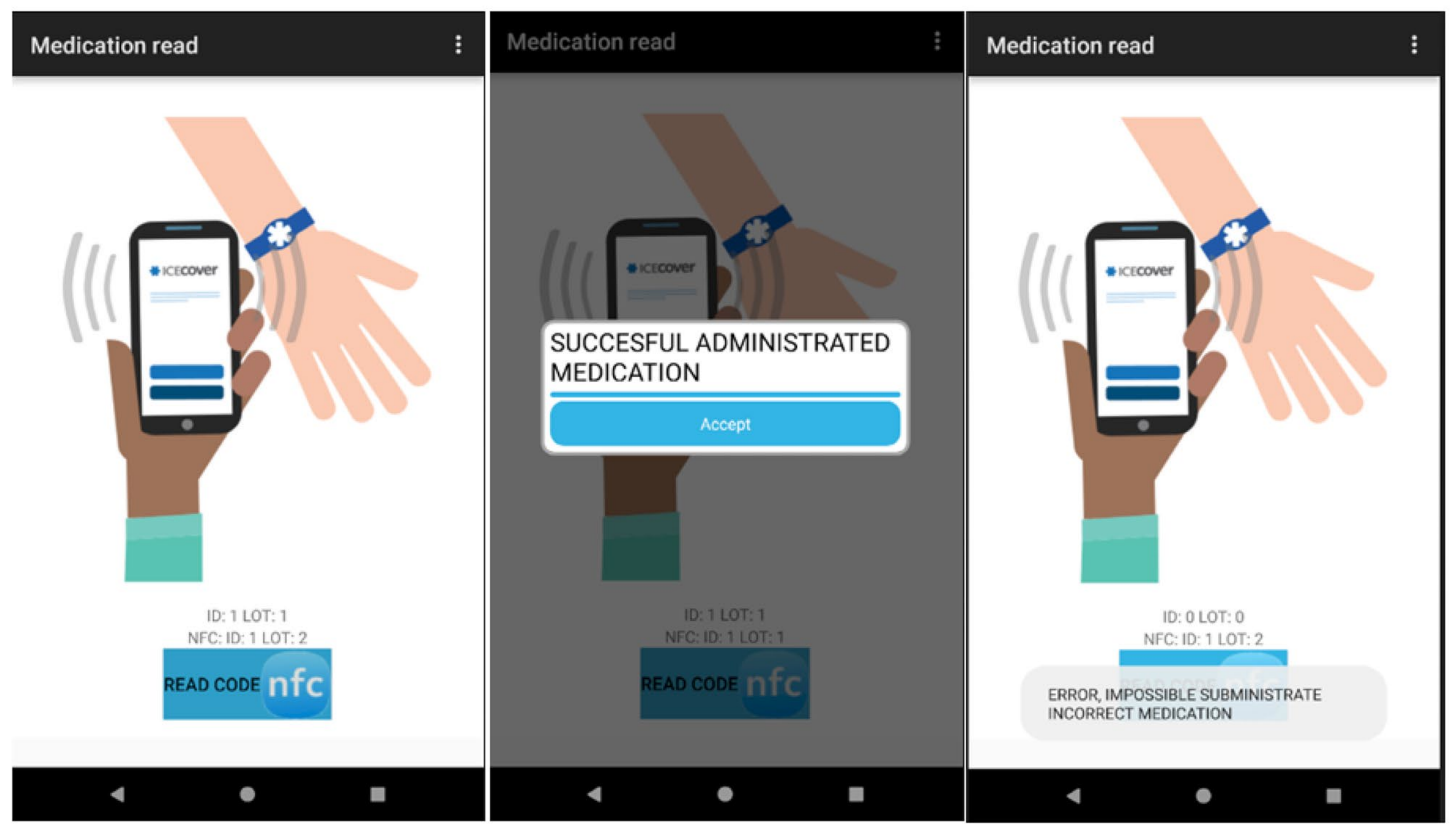
RFID system for tracking in the ICU (source: https://www.mdpi.com/1424-8220/18/5/1627/htm#)

The developed system consists of two subsystems: hardware and software. They have been designed to facilitate the flow of information between all operators involved in the patient care. The administered drug, the healthcare staff and the patient are identified by means of a NFC tag. This tag must be read by the application to obtain the unique identifier (UID) and manage the pending tasks related to the care process of the patient. For example, the application can thus confirm whether a certain drug, prescribed by the doctor, is waiting to be administered to the patient by hospital staff on shift (Fig. 5).

The identification of healthcare staff is important to ensure that each professional profile has access to information based on its category. In a possible scenario, the nursing staff will be able to manage the drugs administration, while the section for drugs prescription is just for physicians. An objective of this system is the possibility of rapidly identifying patients so that it is possible to check which of them have been administered certain batches of drugs, to manage any pharmaceutical alarms.

#### Infection prevention and control

**Fig. 6:**
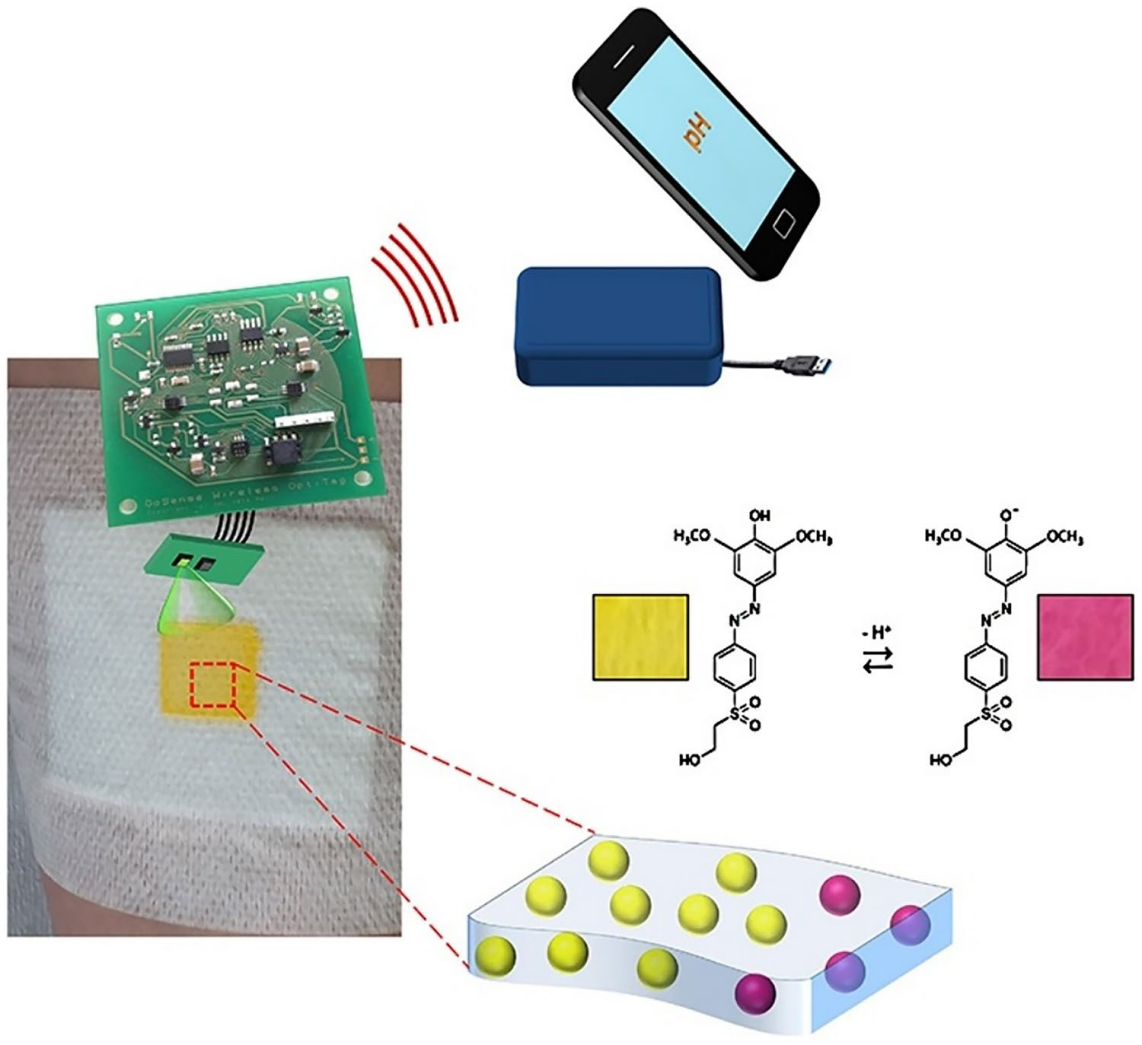
Schematic showing operation of the wireless smart bandage (source: https://www.sciencedirect.com/science/article/abs/pii/S0925400517303222)

It is also important to prevent possible worsening of wounds or infections in time. For example, it is important to monitor the progress of wounds healing to prevent deterioration. It is known that pH is an important biomarker of the state of a wound, normally in the absence of lesions the skin has a slightly acid pH, in the range of 4-6, while when it is damaged this acidic environment is altered. When the wound is acute, pH follows a relatively simple path through a phase of acidic inflammation, followed by a more basic granulation phase, subsequently stabilizing in the 4-6 pH range during re-epithelialization. About the chronic wounds, the process is much more complex [32] and it is very important to monitor this process to get an idea of the progress of the wound, in order to act promptly. To this end it was proposed to fix a pH meter on wound dressings with a non-contact electronic reading based on RFID, through a low-cost optoelectronic interface [29]. Optical measurements are carried out with a wireless sensor framework specifically designed for optical chemical sensing. This framework allows quantitative pH data to be self-measured and wirelessly transferred via RFID to a computer. The system is based on a commercial integrated circuit, the MLX90129, which provides wireless communication functions (RFID and NFC). The optoelectronic sensor consists of an LED light source and a photodiode that measures the light reflected by the pH-sensitive film. The LED and photodiode are controlled by the wireless platform during sample acquisition with an adjustable sampling rate (Fig. 6).

Among the many wireless technologies available, the use of RFID for wound detection is particularly appealing, owing to inherent characteristics such as low power consumption, which allows for longer measurements, or its compatibility with NFC, which allows for data transfer and analysis directly from a smartphone.

**Fig. 7:**
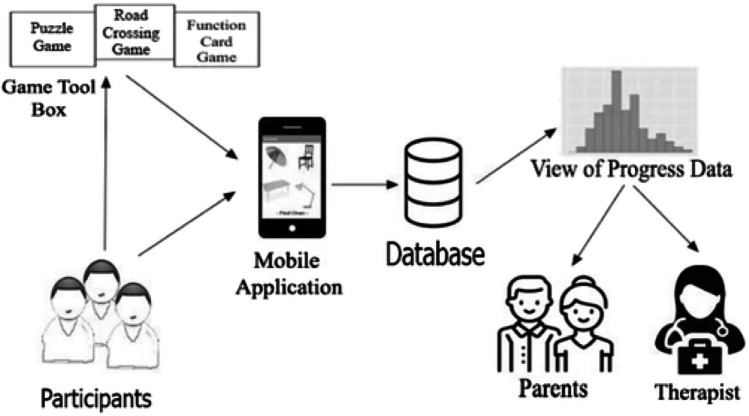
Conceptual design of the proposed gaming tool (source: https://journals.sagepub.com/doi/abs/10.1177/07356331211067725)

The usage of a humidity sensor for diapers is especially important for non-self-sufficient persons, children, or people with certain diseases, who, if not examined often, are vulnerable to skin rashes and bacterial infections [25]. The low-cost smart diaper features a passive RFID tag made of SAP (Super Absorbent Polymer), a subclass of hydrogel that is responsible for the majority of absorption and boosts conductivity when wet. This characteristic is utilised for detection as well as an antenna element in the tag’s construction. The plan was to create a bow-tie antenna made of metal and SAP that expands when wet, increasing the power given to the RFID tag chip. The RFID reader, when placed within the tag’s reading range and linked to the internet, allows you to send a notice to the mobile device associated with it, notifying the healthcare personnel or caregiver in the event of an emergency. Another essential protection is that linked to epidemics; there are crucial steps to be done to avoid catching the virus. So, even when we talk about COVID-19, we know that the WHO has standards in place to attempt to restrict its spread. They are basic principles that must be followed in order to protect ourselves and others; consequently, they must be taught and mastered even by youngsters, but this may be challenging when dealing with autism. Indeed, autistic children’s learning processes are hampered from early childhood due to a diminished inclination to watch and copy others, as well as trouble interpreting others’ words and activities [33]. Technology and gaming, such as the creation of an IoT-based gaming platform, can be beneficial [23]. The platform is made up of three games and comprises of a physical device and a mobile application. To save data, the mobile application is wirelessly connected to the device and the server. Children’s interactions and activities with the device are assessed and saved on the server, allowing past data to be obtained, examined, and analyzed by the server via this application, allowing them to monitor their learning progress (Fig. 7).

A power supply turns on the hardware. There are three switches that correlate to the device’s three games. Only two of these focus on learning Covid-19 infection prevention strategies. The linked gadgets are powered when the corresponding switch is switched on.

**Fig. 8:**
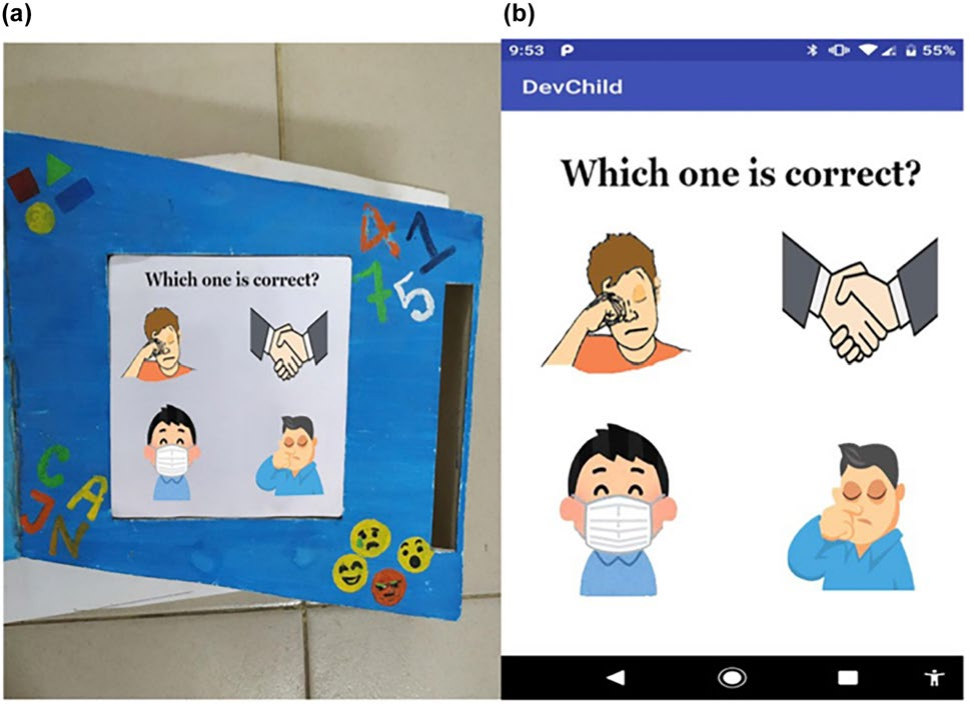
**a** Function card game box and **b** respective app interface

One of these games consists of cards, with each card containing a multiple-choice question and four potential pictures illustrated below. Each card has four piezoelectric sensors and an RFID tag that uniquely identifies it. When a card is placed in the corresponding location of the game box, the system reads the RFID tag associated to the card, allowing it to be viewed on the mobile application, which records the replies in the database. As shown in Fig. 8, the card’s job is to educate a youngster which behaviors are appropriate and which are wrong or to avoid in order to protect us from COVID-19.

A second game is to teach the kid the proper sequence for proper hand hygiene, always in relation to viral transmission prevention. The game is organized by six cards, each of which has a picture and an RFID tag (the tag is used to uniquely identify the cards), which must be placed in the correct sequence on the game box by the kid. The latter is made up of RFID scanners, which will uniquely identify the cards and relay the data to the mobile application. Unfortunately, given the rapid transmission of some viruses, such as COVID-19, we know that preventative measures are sometimes insufficient. Several research have attempted to discover a feasible answer to contact traceability [26]. In one research, an IoT-based approach that gathers information from moving objects is offered [26]. This information is recorded anonymously until bearers test positive for an infectious illness, such as COVID-19, according to the model.

**Fig. 9:**
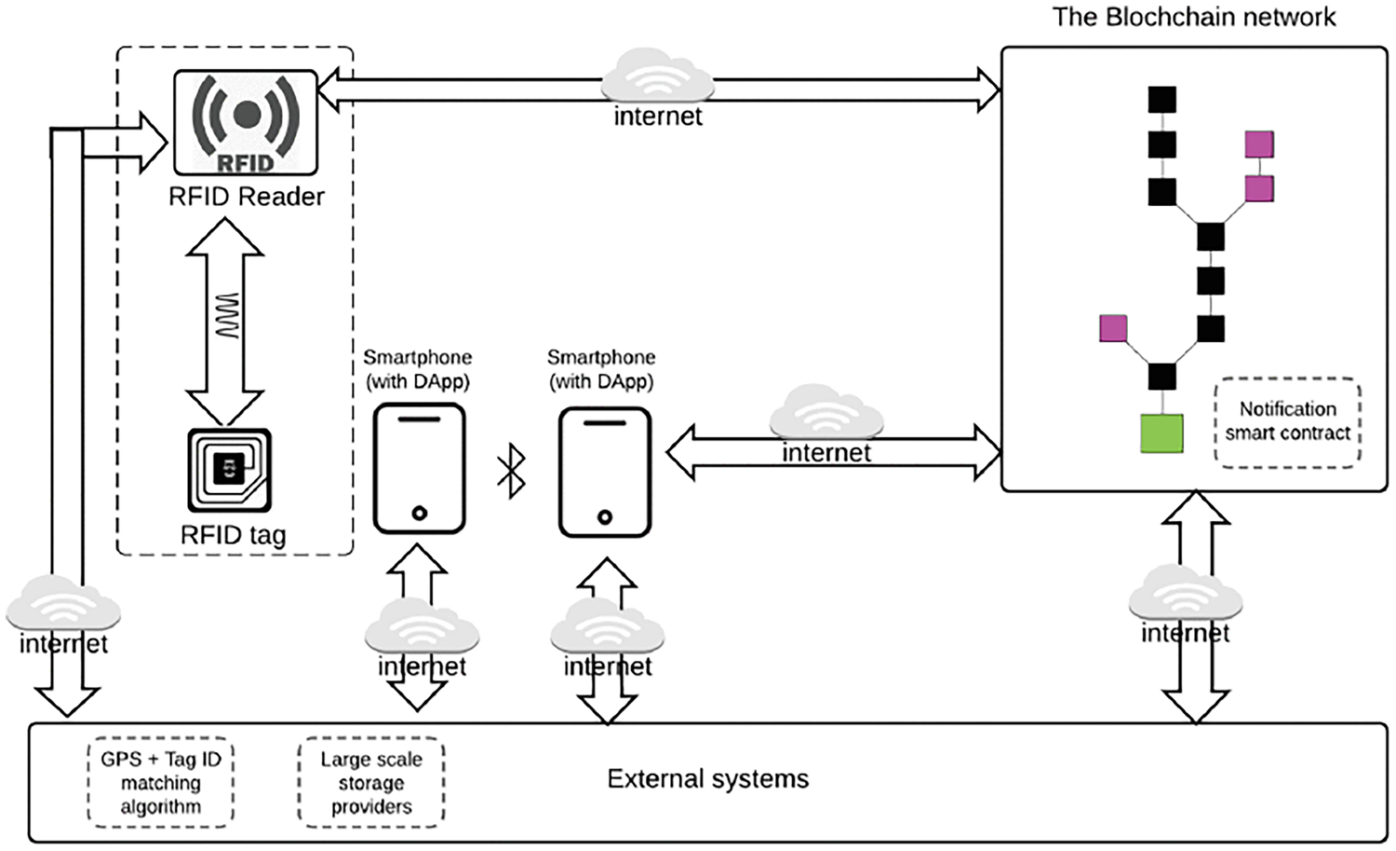
RFID device data flow diagram to the blockchain (source: https://ieeexplore.ieee.org/document/9181512)

The visual architectural model shown in Fig. 9 depicts how data flows from the RFID tag, to the reader, and finally to the blockchain; similarly, proximity data collected by the application downloaded on a mobile device (consider the various applications that were freely downloaded during the Covid-19 pandemic, which were used to detect and prevent any infections), from the geolocalizer of contacts incorporated into it, flow into the blockchain via the Internet. In order to maintain anonymity, the data obtained in this manner is kept using the blockchain. Indeed, because to its qualities and the manner in which data is maintained, it is frequently seen as an alternative to other types of databases for registers administered by public bodies in terms of security, dependability, openness, and prices). The contact geolocalizer is a component of the application (DApp), i.e. the front-end through which users interact with the program. If a citizen with the mobile device or RFID tag is diagnosed with COVID-19 or another infectious disease, the information collected may be utilized to send notifications to contacts.

The collected data is saved on the applicable Smart Contract (SC). A smart contract is a specific collection of instructions recorded on the blockchain that may self-execute activities based on a set of pre-programmed criteria; all of this in an immutable, transparent, and entirely secure manner. To prevent the excessive use of data and the phone battery, information on position changes will be acquired every 10 minutes, as will uploading to the blockchain every twenty minutes. Because the RFID tag lacks the ability to connect to other devices, its contact with other similar devices will be determined by the timestamp information (the timestamp can be defined as a “timestamp,” which is a sequence of characters representing a date and / or a time to determine the actual occurrence of a certain event).

#### Protection measures

**Fig. 10:**
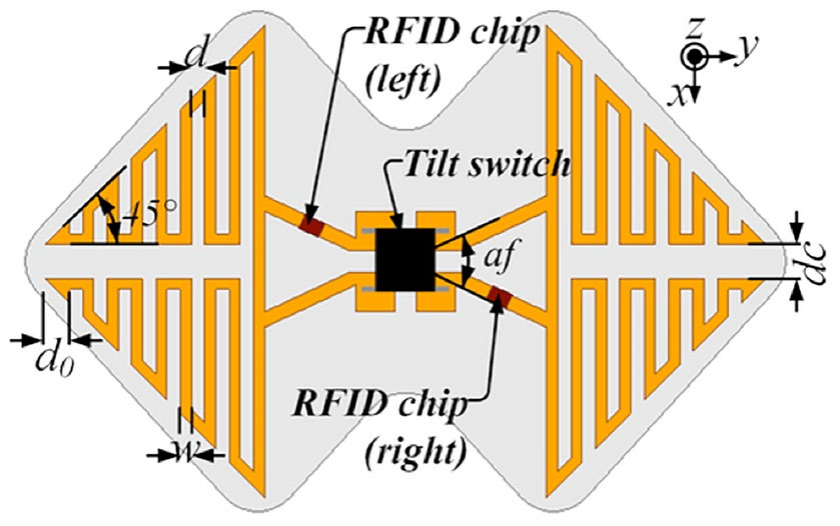
Geometry and photograph of the proposed rolloversensor (source: https://onlinelibrary.wiley.com/doi/abs/10.1002/mop.32648)

The scarcity of health providers is a severe socioeconomic issue in many nations, especially given the aging of the population [34]. Cutting-edge medical technology, like as intelligent wheelchairs, can assist the elderly in living independently, therefore alleviating the shortage of health care. However, the lack of a caregiver makes wheelchair accidents more perilous; rollover is one of the most prevalent, and the following fall of the user is possibly lethal. As a result, an RFID-based rollover monitoring sensor attached to wheelchairs can be quite useful [24]. The suggested sensor is made up of two symmetrical, meandering dipole antennas on the left and right sides, as well as a four-port switch, tilt detector (RBS100600 ONCQUE) in the middle (Fig. 10).

The rollover sensor is intended to be mounted horizontally beneath the wheelchair seat. When the latter is flipped over, the two pins of the tilt switch on the opposite side (right or left) are connected, and the RFID chip on the same side is activated, generating a voltage, by the energy of the signal sent by the RFID reader. Thus, the wheelchair’s protective measures can be activated to minimize injuries by connecting their circuitry to the RFID chip. When this is activated, a response signal containing the chip code is delivered to the reader. In this manner, the RFID-based location algorithm can get the sensor position, and the emergency signal comprising the sensor location will be transmitted to local hospitals or rescue stations, as well as family members.

#### Vital signs monitoring remotely and in real time

**Fig. 11:**
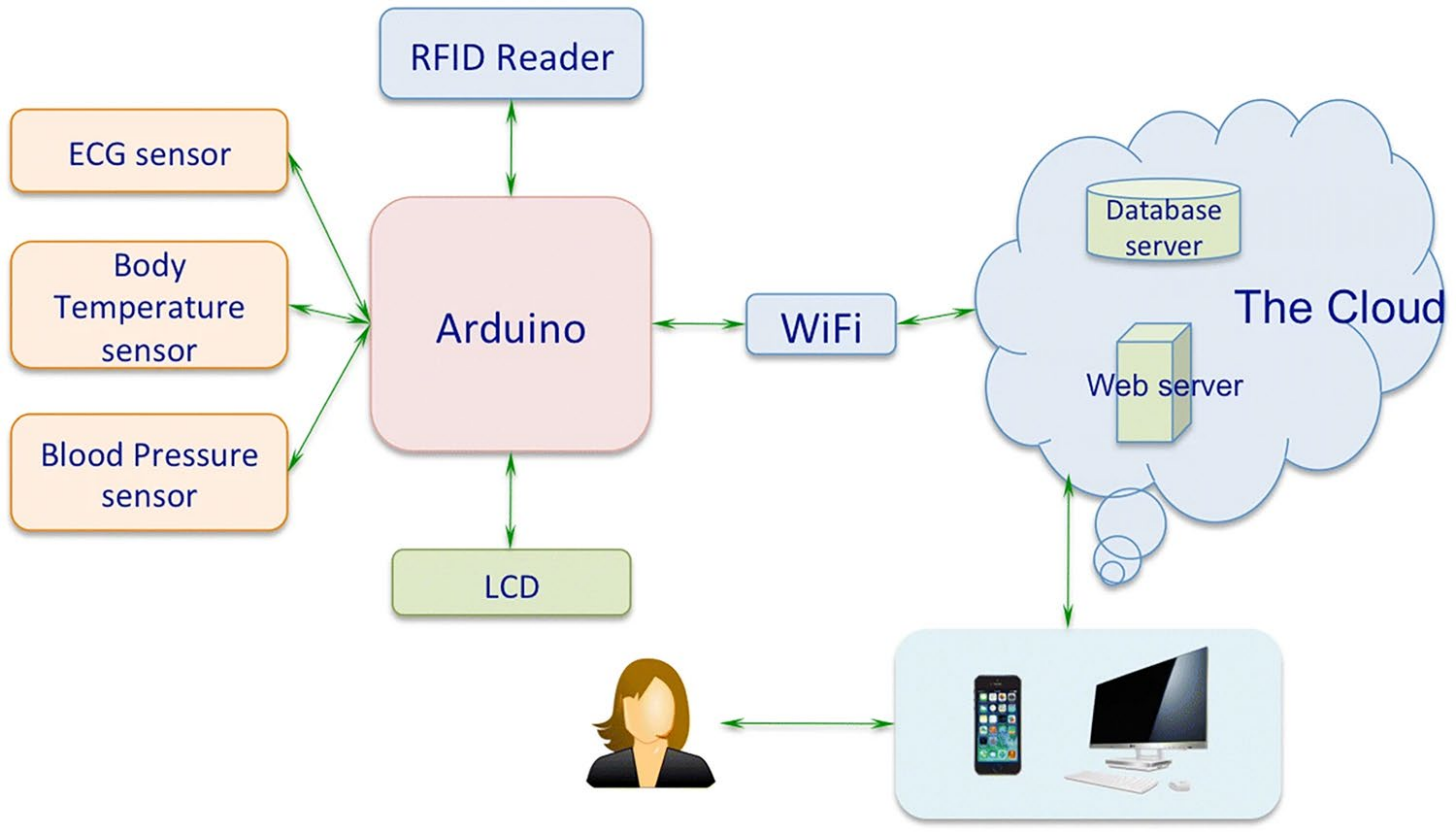
WISE system (source: https://jwcn-eurasipjournals.springeropen.com/articles/10.1186/s13638-018-1308-x)

This is the case with the development of the Wearable IoT-cloud-based hEalth (WISE) system [20], which employs a network of indestructible sensors to monitor the health of people with chronic diseases such as heart disease, diabetes, and Alzheimer’s disease. It is possible to get a number of biomedical signals, including arterial blood pressure, heart beat, blood pressure, and body temperature. WISE was developed on the basis of the hardware platform Arduino, and is integrated with sensor nodes such as the non-invasive sensor designed to measure blood pressure. The connection of an RFID reader to the Arduino platform makes it easier to identify different users. Furthermore, WISE has a WiFi module that allows data to be sent to the cloud, allowing authorized users to access data in real time from any location and at any time. As a result, the WISE system consists of three key components: the WISE body area network (W-BAN), the WISE cloud (W-Cloud), and the WISE users. Connecting the RFID reader to the Arduino platform makes it easier to identify different users. Furthermore, WISE has a WiFi module that facilitates data transfer to the cloud, allowing authorized users to view data in real time from any location and at any time. As a result, the WISE system is made up of three main components: the WISE body area network (W-BAN), the WISE cloud (W-Cloud), and the WISE users (Fig. 11).

W-BAN data may be effectively and efficiently saved and processed in the cloud. To detect and diagnose probable cardiac disease, key characteristics can be extracted. If an aberrant state is identified, an alert is sent to a designated interlocutor, including a text message to physicians or family members, and a warning is presented on the LCD (Liquid Crystal Display) for the users.

#### Monitoring of medical instruments and drugs

Another important issue is the continuous monitoring of medical instruments and drugs that are essential for patient care, for example, to avoid the stock-out in the inventory. A solution could be the use of an automated system defined as “StocKey^®^ RFID Smart Cabinet” [27]. The medical supplies for the patients’ care and those for the surgical operations are labeled with RFID technology when they are supplied to the hospital. In this way, it is possible to manage expiration dates and automatically schedule reorders. The tags, in fact, identify the product with the lot number, serial number and expiration date. The objects thus identified are kept in a closed cabinet (“Faraday cage”), which allows an accurate view of the medical supplies present in the warehouse. All the inputs and outputs of products, thanks to their RFID tags, are read to be incorporated into the electronic inventory of the cabinet. This system was designed primarily for operating rooms, unlike the IoT-based system [14], which was designed for in-hospital or out-of-hospital pharmacies and mainly for drugs. This system also uses the RFID tag above the drug packages, which are read by an RFID reader placed in the center of the compartment, where they are located. Everything is connected to an LED that alerts the manager of that department if a check for missing or expired drugs is necessary. RFID labeling can also be considered one of the best solutions against drug counterfeiting, because information, such as raw materials, the manufacturer, and the pharmaceutical company, is collected and thus identification is facilitated. This is a very important, because counterfeit drugs pose a significant threat to patient safety and public health and cause heavy losses to each State economy. For example, counterfeit drugs to treat malaria and pneumonia cause an estimated 250,000 infant deaths each year.

## Discussion of results

From the analyzed studies, the use of RFID tags seems to be more promising in two scenarios: the first is in the field of surgical instrumentation, since RFID technology allows continuous monitoring of the instruments used during a surgical operation, such as gauze or instruments: scalpels, electrosurgical adapters, forceps, etc. Therefore, the use of RFID tags benefits the patient, in terms of safety, and the medical and nursing staff in carrying out their related duties. The second scenario is that concerning patients’ identification: a correct identification of the patient helps to reduce errors related to the administration of drugs; a quick identification of the patient is very important in case of emergencies launched by the pharmaceutical companies on a specific batch of a drug that could present anomalies or manufacturing errors. Passive RFID tags seem to be the most used, this is probably due to their lower cost compared to active RFID tags, their small size which makes them more flexible, despite their reading range that is much shorter than that of active ones. Although RFID technology holds great promise for Healthcare, there are several risks or barriers that prevent its implementation, in particular the implementation cost and the need to improve data security constitute obstacles to its use within hospitals or public medical facilities. Indeed, data security is a critical issue, since the protection of privacy and sensitive data currently requires careful attention. Another problem is electromagnetic interference (EMI) which occurs when electromagnetic waves from an electronic device interfere with the operation of another electronic device and cause an unwanted response. Many studies from the authors have assessed these aspects by applying risk analysis techniques as well as by investigating electromagnetic compatibility in real hospital settings [35–39]. The use of these technologies still needs to be tested and experimented on a large scale, as experiments have often been carried out using prototypes, in a limited number of places or on a few people.

In this work, the reviewed papers are academic articles, so the results are useful for analyzing the current development state of academic research but may not be suitable for predicting the actual implementation of RFID technology within medical and healthcare facilities.

## Conclusions

The adoption of RFID technology in Healthcare is growing slowly compared to other areas, despite it is a very valuable tool. The proposed papers have been selected by searching the Scopus database. The presented works show that this type of technology can improve patients’ safety by reducing medical errors, that can occur within operating rooms, such as, for example, the retention of surgical material. It can also be the solution to overcome the problem of the black market in counterfeiting drugs, or as a prevention tool designed for monitoring the state of a wound using “smart bandages”. In the selected papers, issues concerning human limitations and relating consequences are addressed. The consequences are faced and prevented using RFID technology, which provides a prompt solution and an improvement in management, inside and outside the hospitals. As previously mentioned, further research is needed, especially on data management, security, and privacy, given the sensitive nature of medical information.

## Data Availability

Not applicable.
